# Memories of a Beloved Friend. *In Memoriam* of Terry Lee Erwin (1 December 1940 – 11 May 2020)

**DOI:** 10.3897/zookeys.936.54751

**Published:** 2020-05-28

**Authors:** David Kavanaugh

**Affiliations:** 1 Department of Entomology, California Academy of Science, San Francisco, California 94118, USA Californian Academy of Sciences San Francisco United States of America

Terry Erwin and I first met in 1966 at San Jose State University while he was completing his Master's degree under the supervision of J. Gordon Edwards, a noted coleopterist and a great mentor, and I was an undergraduate pre-med student. Terry was the assistant curator of the entomology collection and one day invited me in to see the collection there. In subsequent visits, he soon got me interested in carabid beetles and taught me how to identify them to genus using the key to carabid genera in Arnett’s *Beetles of the United States*. Only later did I learn that the key had been written by George Eugene Ball, a man who was to impact both our lives greatly. When I showed special interest in these beetles, Terry gave me a workspace in the museum and taught me how to make use of the collection.

I remember Terry telling me early on that his parents had named him after Terry Lee, the lead character in a very popular comic strip, *Terry and the Pirates*, which ran in broad syndication from the mid-1930’s through the mid-1970’s. Terry Lee was a man of action, charismatic and fearless, adventurous, and so was Terry Lee Erwin. I always wondered how his parents had been so insightful about the son that he would grow up to be. He was simply a superb field man—tireless, eager, always enthusiastic and upbeat and always ready to try some new technique or tackle a new challenge.

Terry took me on my first collecting trip looking specifically for carabids. We collected along Coyote Creek above Anderson Reservoir just south of San Jose, a place he had visited many times for his thesis project on the *Brachinus* species of California. I remember being so excited at finding a single brilliant green elytron and wondering what beetle it was. Terry simply smiled and said that it was a *Chlaenius* species, probably *C.sericeus*. He didn’t laugh at my naive excitement, a fact that I would come to appreciate after I’d found perhaps my thousandth specimen of that species. Many times over the years, I’ve seen him show other novices the same kind of gentle support and respect.

**Figure:**
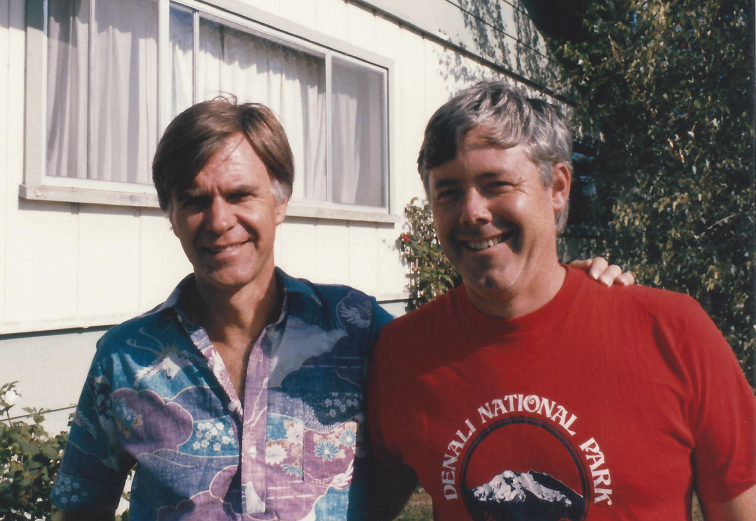
Terry Erwin (left) and Dave Kavanaugh (right) in Petaluma, California, mid 1980’s.

In late summer of 1966, Terry was off to Edmonton, Alberta to work for his PhD with George Ball at the University of Alberta. We kept in contact by mail, which, before email or texting, was a frustratingly slow process, usually involving many days or even weeks for a turnaround of exchanges. He encouraged me from afar through completion of my bachelor's degree and then during my first two years in medical school at the University Colorado in Denver. I kept collecting carabids as a hobby in my spare time and Terry generously identified the many specimens I sent him. Among them were specimens of a new species of *Brachinus*, the group he continued to study for his PhD dissertation project. He ended up naming it *Brachinuskavanaughi*. Terry knew how to encourage and support students and colleagues in many different ways.

Terry didn’t like cold climates at all and this, I think, along with his typical demonically hard work, helped him to complete his dissertation project in less than three years, a record time for a George Ball student. With PhD in hand, he went home to California to pick up items he had left with his parents in Vallejo, loaded up his car, jammed full to the ceiling inside and until the springs were fully compressed, and headed east to begin his postdoc with Philip Darlington, Jr. at the Museum of Comparative Zoology at Harvard. We had arranged to meet at Capitol Reef National Monument (now a national park) at around dinnertime. I was so eager to see Terry again after three years of long-distance correspondence that I left Denver heading west at about 3:00 am. When I realized that I would be way too early if I proceeded straight to Capitol Reef, I decided to make a detour south along the north side of the Colorado River toward Bullfrog. The detour led me to a place called Indian Gulch, just on the southeast edge of the Boulder Mountains in Garfield County, Utah. I stopped to collect briefly and found specimens that would turn out to be a new species, which I later named *Nebriadesolata* for the forsaken place in which I had found them. Unfortunately, I had car trouble and didn’t arrive in Capital Reef until almost midnight. When I got there, I found Terry still waiting for me, sitting at a camp table sorting specimens by Coleman lantern. We shared our experiences of the day, which for him included collecting specimens of *Nebriazioni* Van Dyke, a species at that time known only from the type series. After we swapped a few specimens of each of our *Nebria* species, Terry got up, went into his tent and came out with a bound copy of his massive PhD dissertation on brachinines that he gave me. It was a wonderfully thoughtful, generous and unexpected gesture on his part. This eventful day would lead me to leave medical school and pursue a PhD with George Ball in Edmonton, and also it initiated my career-long passion for nebriine carabid beetles.

Eventually, Terry and I were to hold very comparable positions as curators of beetles in two of the greatest entomological collections in the world. Over 54 years, we remained close friends and confidants. We co-authored ten scientific papers, one published abstract, and two electronic publications together and had several other projects planned or in progress. We also co-supervised three graduate students. In September, 1984, Terry brought me with him to his beloved Amazon rainforest, to Tambopata Reserve in Madre de Dios, Peru, where I helped him with his canopy fogging project there. Keeping up with Terry in that heat and humidity and at the break-neck pace of his work schedule was hard, but I enjoyed it thoroughly. Without experiencing the work first-hand, I could never have appreciated fully the monumental effort that Terry’s fogging work at various tropical rainforest sites over the years represents. He persevered in these ambitious projects over decades and, in the process, generated literally millions of forest canopy specimens that otherwise might never have been seen and which have formed the material basis for dozens of research projects by students and colleagues in addition to his own. Sadly, that trip in 1984 was our only extended time together in the field…my research interests were in much colder places than Terry’s.

Terry was in innovator at heart, always trying new things. When he encountered a problem, he tried to find a simple way around it. For example, his fogging program was his solution to finding out just what lived up in the tropical rainforest canopy, completely out of reach for earth-bound collectors. It was simple, relatively inexpensive and mobile, all attributes that rainforest canopy ziplines, walkways and other fixed structures do not share and certainly much safer than climbing the trees. Another example is Terry’s initial and continued involvement in *ZooKeys*. He saw this as a solution to the taxonomic publication bottleneck that had challenged him and all of us throughout our careers. That solution has worked out pretty well too. See https://zookeys.pensoft.net/article/7316 for a biography of Terry, written in celebration of his 75^th^ birthday and his role as Editor-in-Chief of *ZooKeys*.

Our relationship was much more than just professional. We got to know each other's families well but had all too infrequent home visits due to our bi-coastal disjunct distribution. Terry honored me by asking me to be best man at his wedding to Grace Servat, his loving and accomplished spouse. Although Terry and I had very different habitat preferences, working styles, family lives and strengths, together we covered a lot of ground, complemented each other and made a great team. No other person influenced my career in so many pivotal ways. Although my history with Terry Erwin is unique in many ways, I’m sure it is just one of many such stories those fortunate enough to have met and engaged with him could tell. I mourn his sudden and unexpected passing and I will miss him always.

